# Synergistic Effect of Perineural Dexamethasone and Dexmedetomidine (Dex-Dex) Prolong Analgesic Effect of a Preoperative Interscalene Block

**DOI:** 10.7759/cureus.9473

**Published:** 2020-07-30

**Authors:** Nazir A Noor, Ivan Urits, Omar Viswanath, Alan D Kaye, Jonathan Eskander

**Affiliations:** 1 Anesthesiology and Critical Care, Mount Sinai Medical Center, Miami Beach, USA; 2 Anesthesiology, Critical Care, and Pain Medicine, Beth Israel Deaconess Medical Center and Harvard Medical School, Boston, USA; 3 Pain Management, Valley Pain Consultants - Envision Physician Services, Phoenix, USA; 4 Anesthesiology, Louisiana State University Shreveport, Shreveport, USA; 5 Anesthesiology and Pain Medicine, Portsmouth Anesthesia Associates, Portsmouth, USA

**Keywords:** interscalene brachial plexus block, dexmedetomidine, dexamethasone, dex-dex

## Abstract

The brachial plexus is often a target of regional anesthesia for procedures involving the upper extremities. These include the supraclavicular, infraclavicular, interscalene, and axillary blocks. The cases we present involve the use of an ultrasound-guided interscalene block using 20 mL 0.2% ropivacaine with dexamethasone and 25 mcg dexmedetomidine as the injectate. This particular block technique has proven to be a very useful adjunct to the perioperative anesthetic care and enhanced recovery after surgery (ERAS) protocol for these patients. The series of cases we present include patients receiving the dexamethasone and dexmedetomidine (Dex-Dex) combination in their local anesthetic injectate for the ultrasound-guided interscalene block. Two of the patients underwent arthroscopic shoulder procedures and one underwent a shoulder total arthroplasty with biceps tenodesis. None of the patients required any postoperative opioids for analgesia. Though the technique is fairly new, with only a limited number of case studies described its efficacy, the understanding of the benefits of ERAS has helped it gain some traction in the field of regional anesthesia. Conduction of further large clinical trials is the next step in providing a better understanding of the Dex-Dex adjuvant method as it moves towards becoming a commonly used component of ERAS protocols in the perioperative period.

## Introduction

The interscalene block is one of the many regional anesthetic techniques applied to the brachial plexus. The technique is used for procedures on the distal clavicle, shoulder, and proximal humerus. A successful interscalene block covers the majority of the brachial plexus, sparing the ulnar nerve, which is derived from C8-T1 [1]. With the shift towards an increased implementation of the enhanced recovery after surgery (ERAS) protocol in such operations, the interscalene block proves very useful. However, ropivacaine and bupivacaine, the two commonly used local anesthetics for this block, have a median duration of analgesia of 11.8 hours and 14.8 hours, respectively. Cummings et al. found that the sole addition of dexamethasone to either local anesthetic improved the block of ropivacaine to 22.2 hours and of bupivacaine to 22.4 hours, while the addition of ultrasound guidance or nerve stimulation did not demonstrate any significant improvement in block duration [2]. The use of dexmedetomidine as the sole adjuvant has resulted in similar findings of prolonged block duration. A newer approach that currently has very limited literature is the combination of dexamethasone and dexmedetomidine (Dex-Dex) as the adjuvant to peripheral nerve blocks (PNB) [3,4]. In their prospective randomized study, Zhang et al. demonstrate the prolongation of analgesia with use of a Dex-Dex combination adjuvant to ropivacaine in intercostal nerve blocks [5]. Given the limited literature, aside from a few case reports of different PNB and the previously mentioned prospective study, we present a case series of three patients, with their consent, who all received an interscalene block under ultrasound guidance for procedures involving the shoulder and proximal upper extremity.

## Case presentation

Case 1

**Figure 1:**
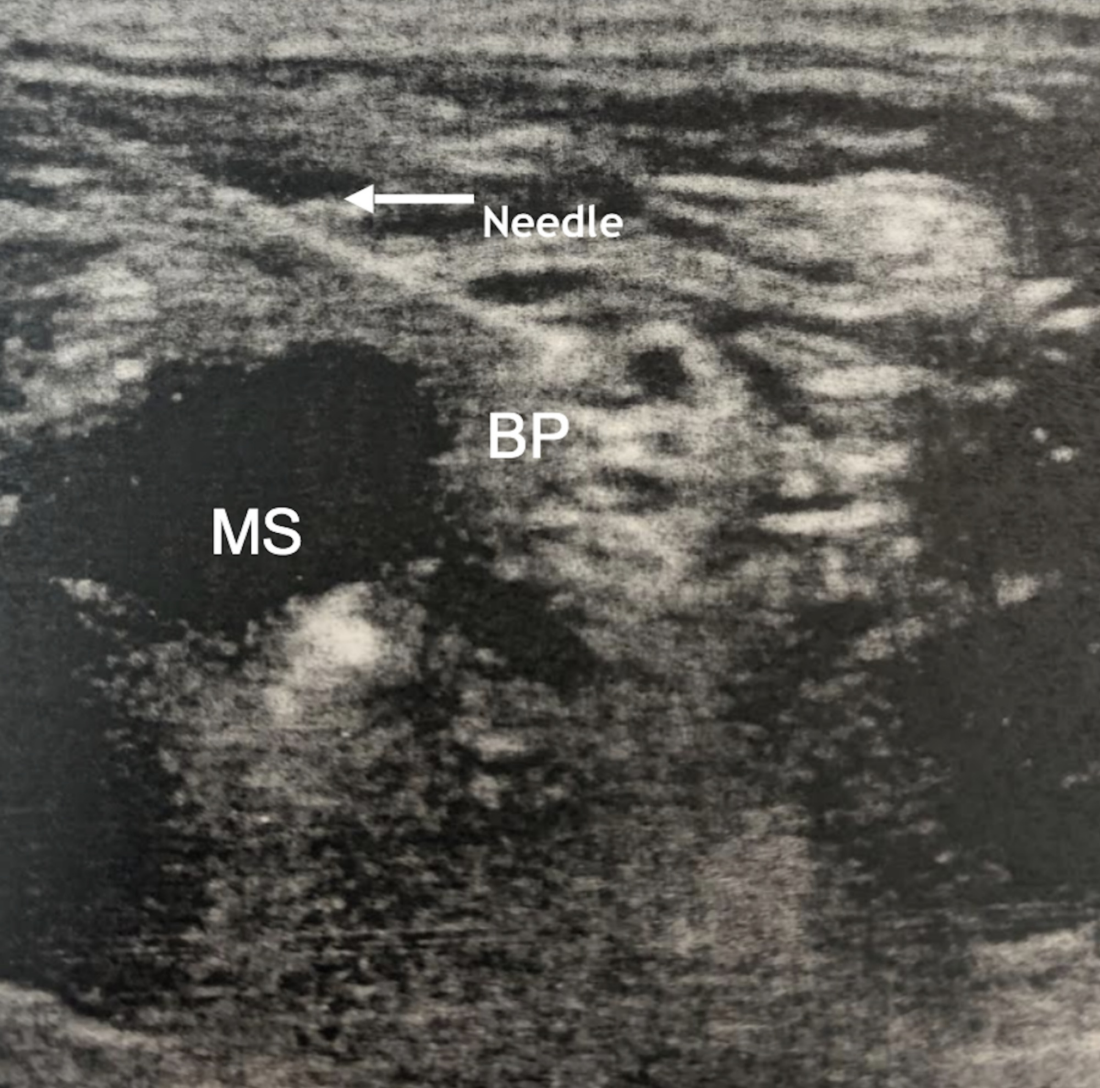
Interscalene block demonstrating brachial plexus roots C5, C6, and C7 (BP) adjacent to the middle scalene muscle (MS)

Our first case was a 27-year-old African-American woman status post motor vehicle accident with chronic low back pain and shoulder pain scheduled to undergo a left shoulder arthroscopic distal clavicle excision. A preoperative ultrasound-guided interscalene block was performed to good effect (Figure 1). The local anesthetic injectate consisted of 20 mL of 0.2% ropivacaine with preservative-free dexamethasone and 25 mcg dexmedetomidine. Her intraoperative course was uneventful. The only postoperative pain the patient experienced was posterior muscle soreness, which she rated a 6/10. She denied any incisional or other pain. Her duration of analgesia lasted for over 72 hours after the procedure with the Dex-Dex adjuvant added to the PNB injectate. It is important to note that her postoperative period did not necessitate any use of opioids. Additionally, no adverse outcomes were experienced.

Case 2

Our second case was a 54-year-old Caucasian woman with a history of hypertension (HTN), hyperlipidemia, and right shoulder pain and stiffness which was diagnosed as adhesive capsulitis undergoing right shoulder arthroscopic capsular release and biceps tenotomy. She received a preoperative ultrasound-guided interscalene block. The injectate concoction comprised of 20 mL of 0.2% ropivacaine with preservative-free dexamethasone and 25 mcg dexmedetomidine. Her intraoperative period was uneventful. Postoperatively, she stated that she had 0/10 pain. The patient experienced over 72 hours of analgesic blockade with the Dex-Dex adjuvant. She did not require any opioids throughout her postoperative period nor did she experience any adverse outcomes directly attributed to the regional anesthetic technique used.

Case 3

Our third case was a 50-year-old Caucasian woman with a history of HTN, chronic obstructive pulmonary disease (COPD), obstructive sleep apnea, morbid obesity, and degenerative joint disease of the left shoulder scheduled to undergo a left shoulder total arthroplasty and biceps tenodesis. As in the previously mentioned cases, the injectate for the interscalene block, completed under ultrasound guidance, was 20 mL of 0.2% ropivacaine with preservative-free dexamethasone and 25 mcg dexmedetomidine. The patient’s intraoperative period was uneventful. In the post anesthesia care unit, she rated her pain a 0/10. The interscalene block with the Dex-Dex adjuvant provided greater than 72 hours of postoperative analgesia. A postoperative opioid-sparing approach was successfully achieved with this technique. She did not experience any adverse outcomes attributable to the use of the Dex-Dex regional anesthetic technique.

## Discussion

The three cases we presented illustrate the synergistic effect of dexamethasone and dexmedetomidine as a combined adjuvant to the local anesthetic injectate in interscalene blocks. As mentioned previously, Cummings et al. described an improvement with the addition of either dexamethasone or dexmedetomidine to the local anesthetic injectate; they were unable to demonstrate a significant difference in postoperative analgesia between the two adjuvants [2]. However, Zhang et al., in their prospective randomized study, demonstrated a significantly prolonged analgesic effect when using the Dex-Dex adjuvant technique for the intercostal nerve block [5]. Our three cases achieved similarly prolonged perioperative analgesia, but a notable difference is the type of regional block we provided our patients, which was the interscalene block in order to provide sufficient regional anesthesia to the site of surgery. But the Dex-Dex method has been reported in cases where a transabdominis plane block is performed, thus supporting the notion that the Dex-Dex combination provides prolonged and improved perioperative analgesia regardless of the type of PNB performed [3,4]. Furthermore, to delve into the mechanism by which dexmedetomidine and its analgesic properties when added to the PNB injectate, Kroin et al. in their 2004 publication in Anesthesiology described the mechanism of action of alpha-2-agonists, such as dexmedetomidine, in regards to improved analgesia and duration of blockade, as being a result of inhibition of the transmission of nociceptive fibers via hyperpolarization-activated cation currents [6]. The pharmacologic mechanism of dexamethasone as an adjuvant to PNB injectate in regards to improved analgesia and increased blockade duration is less understood. Nonetheless, Hewson et al. postulated that it is likely a direct result of its anti-inflammatory properties [7]. And certainly, less inflammation can translate to reduced pain sensation.

## Conclusions

Though the Dex-Dex adjuvant technique is still in its infancy, the synergistic effects of the combination in PNB, and in our case the interscalene block, are becoming reproduced more frequently. However, the literature is still sparse on the use of this technique. These novel cases are a testament to the efficacy of the use of a combination of dexamethasone and dexmedetomidine. Further large clinical trials may provide a much better understanding of combined dexamethasone and dexmedetomidine as adjuvants to the local anesthetic as injectate in regional anesthesia. This will likely serve as an effective and favorable component of ERAS strategies in operations of the upper extremities, specifically those of the shoulder and proximal upper extremity. In turn, we will be taking a step in the right direction towards further improvement in analgesia, duration of blockade, reduction in opioid use, length of hospital course, and other perioperative complications.
