# Correction: Synergistic Effect of Perineural Dexamethasone and Dexmedetomidine (Dex-Dex) Prolong Analgesic Effect of a Preoperative Interscalene Block

**DOI:** 10.7759/cureus.c40

**Published:** 2020-11-16

**Authors:** Nazir A Noor, Ivan Urits, Omar Viswanath, Alan D Kaye, Jonathan Eskander

**Affiliations:** 1 Anesthesiology and Critical Care, Mount Sinai Medical Center, Miami Beach, USA; 2 Anesthesiology, Critical Care, and Pain Medicine, Beth Israel Deaconess Medical Center and Harvard Medical School, Boston, USA; 3 Pain Management, Valley Pain Consultants - Envision Physician Services, Phoenix, USA; 4 Department of Anesthesiology, Louisiana State University Shreveport, Shreveport, USA; 5 Anesthesiology and Pain Medicine, Portsmouth Anesthesia Associates, Portsmouth, USA

This article was originally published with the fourth author, Alan D. Kaye, missing his middle initial. The author's name has been corrected to include the middle initial. Cureus regrets the error.

